# Progress in detecting cell-surface protein receptors: the erythropoietin receptor example

**DOI:** 10.1007/s00277-013-1947-2

**Published:** 2013-12-14

**Authors:** Steve Elliott, Angus Sinclair, Helen Collins, Linda Rice, Wolfgang Jelkmann

**Affiliations:** 1 Department of Hematology/Oncology, Amgen Inc., One Amgen Center Drive, Mail Stop 15-2-A, Thousand Oaks, CA 91320 USA; 2 Department of Medical Writing, Amgen Inc., Thousand Oaks, CA USA; 3 Institute of Physiology, University of Lübeck, Lübeck, Germany; 4 Present Address: Gilead Sciences, Inc., Foster City, CA USA

**Keywords:** Erythropoietin receptor, Erythropoiesis, Cancer, Angiogenesis, Antibody

## Abstract

Testing for the presence of specific cell-surface receptors (such as EGFR or HER2) on tumor cells is an integral part of cancer care in terms of treatment decisions and prognosis. Understanding the strengths and limitations of these tests is important because inaccurate results may occur if procedures designed to prevent false-negative or false-positive outcomes are not employed. This review discusses tests commonly used to identify and characterize cell-surface receptors, such as the erythropoietin receptor (EpoR). First, a summary is provided on the biology of the Epo/EpoR system, describing how EpoR is expressed on erythrocytic progenitors and precursors in the bone marrow where it mediates red blood cell production in response to Epo. Second, studies are described that investigated whether erythropoiesis-stimulating agents could stimulate tumor progression in cancer patients and whether EpoR is expressed and functional on tumor cells or on endothelial cells. The methods used in these studies included immunohistochemistry, Northern blotting, Western blotting, and binding assays. This review summarizes the strengths and limitations of these methods. Critically analyzing data from tests for cell-surface receptors such as EpoR requires understanding the techniques utilized and demonstrating that results are consistent with current knowledge about receptor biology.

## Introduction

A current focus of oncology research is identifying tumor-specific antigens, such as cell-surface protein receptors. Therapeutic treatments have been developed to target some of these receptors, and receptor expression is sometimes used as a prognostic indicator. However, detecting the presence of a cell-surface receptor on tumor cells does not guarantee that inhibiting receptor activity will block tumor progression. For example, colorectal tumors overexpressing epidermal growth factor receptor (EGFR) respond to anti-EGFR antibody treatment only in the absence of mutations that constitutively activate *KRAS* [1]. In contrast, overexpression of human epidermal growth factor receptor 2 (HER2) on breast-cancer cells is highly predictive of response to anti-HER2 antibody therapy [2]. Though cancer-treatment decisions and prognosis often depend on knowing whether a protein receptor is present, tests for detecting receptors can be unreliable. For example, inaccurate outcomes from HER2-immunohistochemical tests can occur due to issues with fixation, assay validation, equipment calibration, testing reagents, and interpretation criteria, leading to both false-positive and false-negative results [3, 4]. Because of the uncertainty of some HER2 laboratory testing procedures, guidelines were published to improve testing quality [5].

As new receptors are discovered and targeted therapies developed, a basic understanding of the strengths and limitations of methods for detecting, quantifying, and characterizing cell-surface receptors becomes increasingly important for cancer treatment/prognosis and for interpreting data. Amplified receptors can be identified by detecting increased gene-copy number (via Southern blotting and fluorescence in situ hybridization [FISH]) or increased mRNA levels (via reverse transcriptase-polymerase chain reaction [RT-PCR], Northern blotting, or microarray) (Table 1). Increased receptor-protein levels can be detected in tissue sections (via immunohistochemistry [IHC]), in cell homogenates (via Western blotting), or on the surface of intact cells (via binding assays with labeled-receptor ligand or flow cytometry with specific antibodies). Evaluating the presence of functional protein involves examining if downstream signaling or enhanced growth/cell survival occurs after cell exposure to the receptor's ligand. Any single method requires adequate controls and confirmation of results to exclude false-positive and/or false-negative outcomes.

**Table 1:** Common laboratory techniques for examining the cell biology of a protein receptor

Detection method	Strengths	Limitations
Genomic amplification		
FISH	Localizes defect to cell	Semi-quantitative
	Localizes defect to chromosome	May not correlate with gene expression
Array CGH	Quantitative	May not correlate with gene expression or protein synthesis
	Localizes defect to specific region of chromosome	Population based^a^
Southern blotting	Easy to perform	Semi-quantitative
	Moderately sensitive	Population based
		May not correlate with gene expression or protein synthesis
mRNA analyses		
Northern blotting	Determines transcript size	Insensitive
	Determines potential spliced variants	Time consuming
		Population based
		May not correlate with protein synthesis
RT-PCR	Easy to perform	Population based
	Moderately sensitive	May not detect different spliced forms
	Semi-quantitative	May not correlate with protein synthesis
Q-RT-PCR (Real Time)	Moderately difficult to perform	Population based (unless laser-dissected samples)
	Very sensitive	May not detect different spliced forms
	Quantitative	May not correlate with protein synthesis
Microarray	Easy to perform	Population based
	Moderately sensitive	May not detect different spliced forms
	Quantitative	May not correlate with protein synthesis
	Broad gene profiling	
Protein analyses		
ELISA	Easy to perform	Need well-validated antibodies
	Moderately sensitive	Population based
	Quantitative	May not detect different spliced protein forms
		May not correlate with function
Western blotting	Easy to perform	Need well-validated antibodies
	Moderately sensitive	Population based
	Semi quantitative	May not correlate with protein function
	Protein sizes confirmed	Unable to determine location of expression in a cell
IHC	Moderately difficult to perform	Need well-validated antibodies
	Moderately sensitive	May not correlate with protein function
	Semi-quantitative	May not detect different spliced protein forms
	Determine location of protein expression in cell	
Binding assays	Moderately sensitive	Need well-validated reagents
	Easy to perform	May not correlate with protein function
	Quantitative	May be able to detect different spliced forms
	Individual cell analysis if flow-cytometry based	

*FISH* fluorescence in situ hybridization, *CGH* comparative genomic hybridization, *RT-PCR* reverse transcriptase-polymerase chain reaction, *Q* quantitative, *ELISA* enzyme-linked immunosorbent assay, *IHC* immunohistochemistry

^a^Population based refers to an analysis of multiple cells instead of a single cell

**Fig. 1:**
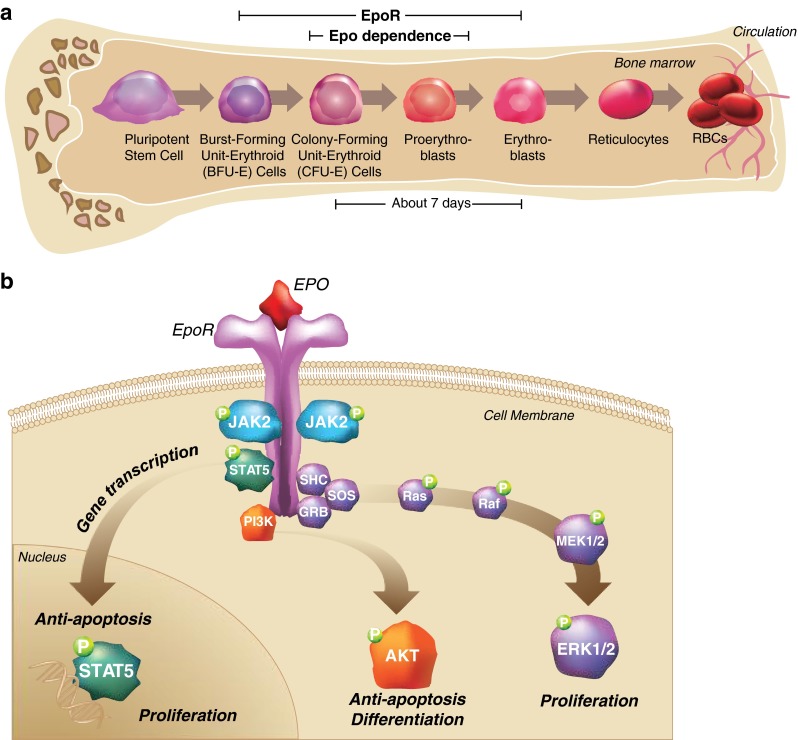
The process of erythropoiesis. Erythroid progenitors in the bone marrow that depend on Epo and EpoR for differentiation into mature red blood cells (**a**). The signaling pathways stimulated by EpoR upon binding to Epo (**b**). *EpoR* erythropoietin receptor, *Epo* erythropoietin, *RBCs* red blood cells. Adapted from *Biologics: Targets and Therapy*, Volume 6, Elliott S and Sinclair AM, The effect of erythropoietin on normal and neoplastic cells, pages 163–89, Copyright (2012), with permission from Dove Medical Press Ltd [6]

To illustrate the strengths and limitations of various methods for detecting the presence, expression, and function of cell-surface protein receptors, we use examples from the literature regarding the cell-surface erythropoietin receptor (EpoR). Normally, EpoR is expressed on erythrocytic progenitors and precursors in bone marrow where it mediates red blood cell production in response to erythropoietin (Epo) produced by the kidneys (Fig. 1) [6]. Some clinical studies have suggested that patients with cancer treated with recombinant human Epo (rHuEpo) or other erythropoiesis-stimulating agents (ESAs) have decreased loco-regional control of tumor growth and/or decreased survival [7]. Potentially explaining these observations, it has been hypothesized that ESAs could bind and activate EpoR on tumor cells to promote their growth and/or survival [8, 9] or stimulate EpoR on endothelial cells to promote tumor angiogenesis [10]. However, other reports indicate that EpoR is not required for normal development of organs or endothelium [11], there is no clinical progression of tumors in response to ESAs, that tumor and endothelial cells do not express functional EpoR, and that some methods of testing for EpoR have led to false-positive results [6, 12–14].

This review summarizes techniques commonly used to identify cell-surface receptors in the context of the large quantity of published EpoR research. The most commonly used technique in clinical care, IHC, will be discussed. Since evaluating the strengths and limitations of other EpoR-detection methods requires background about normal EpoR and erythropoiesis, general information about EpoR biology will be briefly reviewed. Detailed information will then be presented on techniques used to examine specific aspects of EpoR expression, function, and hypothesized roles in tumor progression/angiogenesis. To identify articles for inclusion in this narrative review, several broad searches of the biologic and medical literature were carried out using the Ovid systems (Medline, EMBASE, and BIOSIS Previews).

## Immunohistochemical staining

IHC is a widely used antibody-based test for detecting receptors, such as HER2, in tumors. IHC testing requires an antibody that specifically recognizes the receptor (Table 1). Tissue collection, fixation, and sectioning influence the ability of an antibody to bind a receptor [5]; thus, controls are essential to exclude false-negative and false-positive results. Further, interpretation of results requires training on understanding and quantifying outcomes (e.g., 0–3+ values in HER2 IHC reporting) [5].

**Fig. 2:**
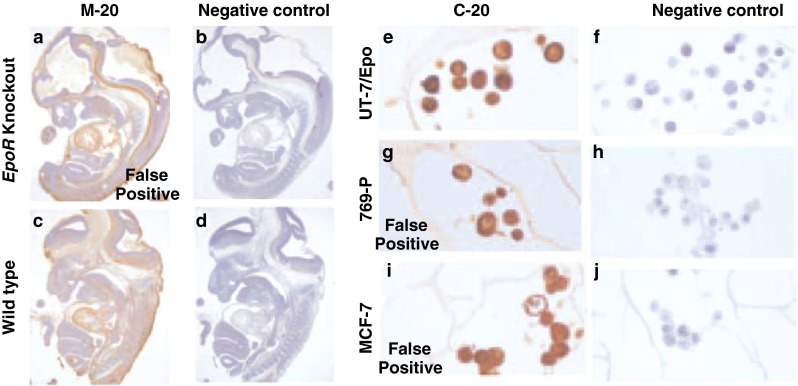
Examples of false-positive IHC using commercial non-specific EpoR antibodies. Wild-type and *EpoR* knockout-mouse embryos stained with non-specific “anti-EpoR” M-20 antibody (**a, c**) and rabbit-IgG antibody (negative control) (**b, d**). IHC in cell lines positive (UT-7/Epo) and negative (769-P, MCF-7) for EpoR using non-specific “anti-EpoR” C-20 antibody (**e, g, i**) and rabbit-IgG antibody (negative control) (**f, h, j**). Adapted with permission: *Blood* 2006;107:1892–1895 [16]

Although published guidelines exist on validating and employing antibodies for IHC, these guidelines are not always followed [15]. With EpoR, many commercially available anti-EpoR antibodies are nonspecific in that they bind to non-target proteins and other cell-matrix structures [16–18]. Notably, there are currently no anti-EpoR antibodies with the sensitivity and specificity to detect EpoR by IHC, yet there are dozens of IHC studies describing EpoR expression based on results with antibodies shown to be nonspecific [6]. For example, an association was earlier noted between staining intensities in head and neck tumor sections (stained with the “anti-EpoR” polyclonal antibody [C-20)] from Santa Cruz Biotechnology Inc.) and the clinical outcomes of the patients who were treated with ESAs [8]. However, several groups of investigators subsequently demonstrated that C-20 produces false-positive signals because it binds to non-EpoR proteins. Furthermore, in other studies, staining by the antibody neither correlated with EpoR expression [19] nor could it differentiate between EpoR-positive and EpoR-negative cells in IHC (Fig. 2) [16–18]. One of the cross-reactive proteins that C-20 binds is heat-shock protein 70 (HSP-70), which was misidentified as EpoR in Western blotting experiments [16]. HSP-70 levels increase with stress-response [20]. Control experiments, using negative-control antibodies of the same isotype or antigen blocking, may eliminate some, but not all, types of false-positive results [14, 15].

Cell lines or tissues expressing/not-expressing EpoR should be used as positive and negative controls, respectively. However, few EpoR-positive cell types are available [21]. EpoR-positive cells also need to express physiologically relevant levels of EpoR protein to be informative. Purified-erythroid precursor cells that express EpoR and are Epo-responsive are difficult to obtain in large quantities. Tissues such as placenta [22] or cell lines such as HeLa [23], MCF-7 [24], or K562 [25], which have been used as EpoR-positive controls, are not Epo-responsive and express EpoR levels greatly below (>100-fold lower) those of known Epo-responsive cells (e.g., erythroid progenitor or UT-7/Epo human-leukemic cells) [21], thereby raising questions about the validity of studies using them. When a specific and sensitive EpoR antibody is applied, EpoR is low to undetectable on immunostaining in non-myeloid cells or tumor cells [14, 26] which is consistent with the low levels of *EpoR* transcripts in those cell types [6, 21].

In summary, IHC is widely used to identify clinically relevant receptors (EGFR, HER2), but proper controls should be employed with all IHC testing to detect both false-positive and false-negative results. Use of IHC to identify EpoR has been hampered by lack of specific EpoR antibodies and of control cell types that clearly express/do not express EpoR.

## Biology of Epo and EpoR

### Physiological role of EpoR

 The inherent problems with many detection methodologies are highlighted by EpoR where confusion exists about its role in tumors. The principal function of the Epo/EpoR system is regulation of erythropoiesis. Epo stimulates erythropoiesis by binding and activating EpoR on the surface of erythrocytic progenitors in the bone marrow (Fig. 1a) [6]. Endogenous Epo is produced primarily in the kidneys and is regulated by oxygen tension. In blood plasma, Epo levels are approximately 10–20 mU/mL (5 pM), but can increase 1,000-fold with extreme hypoxia [6]. Interestingly, neither Epo nor EpoR are required for commitment to the erythroid lineage or for proliferation and differentiation of burst-forming units-erythroid (BFU-Es) to colony-forming units-erythroid (CFU-Es) [27, 28]. However, Epo and EpoR are crucial in vivo for the survival, proliferation, and terminal differentiation of the CFU-Es and their progeny.

Adult human Epo-responsive erythroblasts, which have the highest levels of EpoR, still have relatively low receptor protein levels compared to other receptor types (approximately 100–1,000 cell-surface EpoR molecules per cell). However, EpoR possess a very high affinity for Epo (dissociation constant [Kd] ∼100 pM) [29]. The EpoR density declines as cells differentiate into proerythroblasts. At the normoblast stage, cells stop dividing, extrude nuclei and mitochondria, and become reticulocytes that enter circulation. Reticulocytes and mature red blood cells do not express EpoR and are unresponsive to ESAs [29].

### EpoR transcription and translation

EpoR expression in erythropoietic tissues is not influenced by Epo [28, 30]. Rather, *EpoR*-gene transcription is controlled by erythroid-specific transcription factors, including GATA-1 [31] and stem-cell leukemia (SCL) protein [32], and *EpoR* transcript levels correlate with GATA1 and SCL transcript levels [6]. Thus, cell types lacking GATA-1 and SCL (e.g., those in non-hematopoietic tissues) express lower levels of *EpoR* transcripts [13]. *EpoR* mRNA is translated into a 508 amino-acid protein, which is translocated to the plasma membrane and transported to the cell surface [33, 34]. During this process, a signal peptide is removed and a carbohydrate chain added, resulting in a transmembrane protein with a calculated molecular mass of 56 to 57 kDa [16]. To be Epo-responsive, cells must express EpoR on the cell surface [16]. In cells that express EpoR, however, <10 % of EpoR protein appears on the cell surface; the remainder is degraded into intracellular EpoR fragments that can be detected with specific EpoR antibodies [12, 21, 35, 36].

### EpoR activation and downstream signaling

EpoR protein does not possess intrinsic tyrosine-kinase activity and requires accessory factors (e.g., Janus kinase 2 [JAK2]) for cell-surface transport and downstream signaling [37, 38]. EpoR is activated when a single Epo molecule binds two cell-surface EpoR molecules and effectively “cross-links” them (Fig. 1b) [39–42]. Epo binding induces cross-phosphorylation of EpoR and JAK2 [6, 43]. This activates downstream proteins such as signal transducer and activator of transcription 5 (STAT5), extracellular-signal regulated kinase (ERK), and phosphatidylinositol-3 (PI3) kinase/AKT pathways [44]. Following activation, negative regulators of EpoR down-modulate responses [45, 46].

## Preclinical EpoR studies: from mRNA expression to functional testing

### mRNA expression: PCR, Northern blotting, and microarray

Presence of *EpoR* mRNA is necessary, but not sufficient, for the expression and functionality of EpoR protein. *EpoR* mRNA must be translated into protein that is translocated to the cell surface. These processes involve multiple and limiting molecular factors. Published techniques for detecting *EpoR* mRNA in solid-tumor cells include RT-PCR (including real time quantitative RT-PCR), microarray, and Northern blotting [13, 47, 48]. As with IHC, positive and negative controls are required. The mRNA levels must be evaluated, as sufficient levels are needed for EpoR protein synthesis, surface expression, and function (recall that low levels of EpoR may not be physiologically relevant and further that <10 % of EpoR protein is expressed on the cell surface). RT-PCR is sensitive and can detect low (basal) levels of mRNA transcripts, but at the cost of potentially nonspecific amplification and with questions about the significance of the detection. Northern blotting is less sensitive, but since it uses electrophoreses to separate mRNA samples by size, this parameter can be used to eliminate some false positive data. Microarrays allow for a broad examination of gene expression, but require careful analysis of multiple samples to provide definitive and quantitative results.

The above specified techniques have enabled detection of *EpoR* mRNA in some tumor and normal cells outside the erythroid compartment, but at relatively low quantities (at 10- 1,000-fold lower levels than in positive controls) [6, 13, 19, 26, 47, 49]. Unlike known oncogenes such as *HER2* and *EGFR*, *EpoR* mRNA is not elevated when tumor samples are compared with normal samples [47, 50, 51]. Reports suggesting otherwise were based on non-quantitative or uncontrolled RT-PCR studies [49].

In summary, methods of detecting *EpoR* mRNA are sensitive, but interpreting results requires controls to detect false-positive data combined with quantitative methods to determine if mRNA levels are adequate to produce physiologically relevant amounts of functional EpoR protein.

### Western blotting

**Fig. 3:**
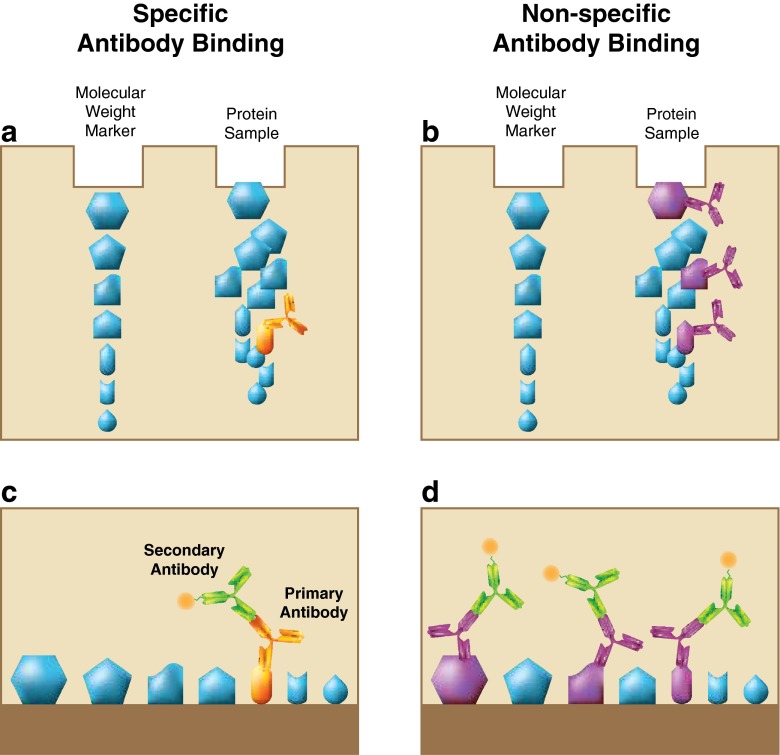
Specific and non-specific antibody binding to proteins on a Western blot. Cell homogenates are separated by size and proteins are detected with antibodies. A specific antibody will bind one particular protein (**a**). A non-specific antibody will bind off-target proteins (**b**). Antibody binding can be detected with a second antibody that emits a signal (**c, d**)

Receptor protein can be detected by tests such as IHC (discussed earlier) and Western blotting. These tests require an antibody that specifically binds the receptor of interest. Western blotting involves separating proteins from a cell homogenate according to size, immobilizing them on a membrane, and using an antibody to detect a specific protein (Fig. 3). Since cell homogenates are used, intracellular and cell-surface proteins cannot be distinguished. Like IHC, Western blotting can involve antibodies that cross-react with non-target proteins, particularly if non-target proteins contain a region with structural similarity to the target protein (Fig. 3). This problem occurs frequently with polyclonal antibodies but even monoclonal antibodies can show nonspecific cross reactivity to non-target proteins, especially when sensitive detection methods are used. Given that EpoR is expressed at low levels, cross-reactivity and false-positive results are particularly problematic with immunologic techniques. Confidence is increased in a positive result if the “band” detected corresponds to the correct size of the target protein. However, false-positive results can occur if a non-specific antibody recognizes a band of the correct size that is not the target protein as has occurred when using commercial anti-EpoR polyclonal antibodies such as M20 from Santa Cruz, Inc. [14].

Western blot methodologies have been employed extensively in attempts to detect EpoR in extracts from tumors and tumor-cell lines. Positive results have been reported, but most came from studies that used antibodies with insufficient specificity/sensitivity to detect EpoR and that only rarely used appropriate control-cell types to exclude false-positive results [16, 49]. Thus, incorrect proteins have been misclassified as EpoR [14, 16].

**Fig. 4:**
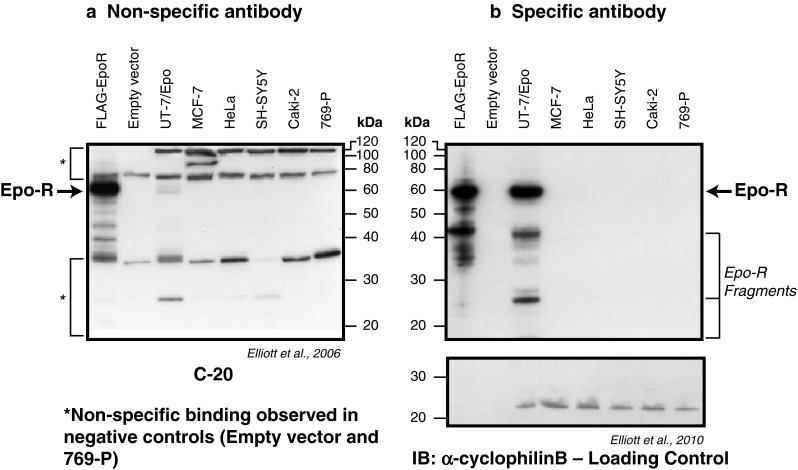
Western blots showing non-specific and specific binding of EpoR antibodies to cellular proteins. The 59-kDa EpoR protein is shown (*arrow*) in positive controls FLAG-EpoR and UT-7/Epo. Nonspecific C-20 antibody binds EpoR and other proteins (*asterisk*) (**a**). Specific-antibody A82 binds EpoR and EpoR fragments but not other proteins (**b**). Adapted with permission: *Blood* 2006;107:1892–1895 [16] and *J. Immunol Methods* 2010;352:126–139 [12]

The full-length EpoR protein migrates at approximately 59 kDa with polyacrylamide gel electrophoresis (Fig. 4) [16]. The commercially available, widely used Santa Cruz polyclonal C-20 EpoR antibody was reported to detect a putative EpoR of 66 kDa (Fig. 4) [16, 18]. However, this 66-kDa protein was identified as the cross-reacting HSP-70 that has some sequence homology to EpoR. When using a specific monoclonal anti-EpoR antibody suitable for Western blotting (such as monoclonal anti-EpoR antibody A82), a 59-kDa EpoR protein can be detected in erythroid cell extracts but not in tumor biopsies [14] or most tumor-cell lines [21]. When EpoR protein was detected in cell lines, protein levels were 10- to 1,000-fold lower than those in cells known to bind or respond to Epo [13, 21].

### Binding assays

The presence of cell-surface receptor protein on live cells can be determined by binding assays with labeled ligand or by flow cytometry with labeled antibodies to the receptor. However, non-specific binding is possible even with high-affinity ligands [52]. In addition, structural changes to the ligands can be introduced through the labeling process, which can increase off-target binding. As with IHC and Western blotting, the specificity and sensitivity of antibodies used for flow cytometry needs to be demonstrated.

Binding assays using labeled forms of Epo or anti-EpoR antibodies with flow cytometry have been used to detect cell-surface EpoR protein. ^125^I-rHuEpo or biotinylated-rHuEpo binding was detected on erythroid and some myeloid-cell types with a dissociation constant (Kd) of ∼100 pM [29, 53, 54]. However, in controlled experiments, no binding of either ^125^I-rHuEpo or of specific anti-EpoR antibodies to non-hematopoietic cells or primary-tumor cells was detected [21, 47]. In a survey of 61 non-hematopoietic tumor-cell lines, only one cell line had detectable (low level) EpoR protein on the cell surface [21]. Other binding studies suggested that EpoR could be detected on some non-hematopoietic tumor-cell lines [55]. In these studies, however, the Kd (10,000–16,000 pM) was 100-fold higher than that in EpoR positive-control cells, suggesting nonspecific binding.

### Functional receptor testing

An alternative to direct measurements of receptors is to examine responses to ligand addition, thereby demonstrating that the receptor is not only present but functional. Such methods can be very sensitive but can also be prone to false-positive results. Determining if a cell-surface receptor is functional requires demonstrating: (1) presence of intracellular-signaling networks responsive to binding of receptor ligand; (2) signaling occurs with relevant concentrations of receptor ligand; and (3) receptor concentrations are high enough to provide relevant signal strength. Similar to direct testing methods, appropriate controls must be used to detect false-positive and false-negative results.

1. Appropriate intracellular-signaling networks must be present in cells for ligand responsiveness.

Even if receptor mRNA or cell-surface proteins are detected, it is still necessary to demonstrate that intracellular-signaling networks are activated in response to the ligand binding the receptor. Demonstrating receptor activation can involve detecting phosphorylation of intracellular signaling-pathway proteins known to operate downstream of the activated receptor in normal, responsive cells. Since ERK and AKT act downstream of EpoR in erythroid cells, activation of EpoR can be assessed by using antibodies that recognize phosphorylated epitopes of ERK and AKT. As noted above, such experiments require demonstration of antibody specificity and appropriate sensitivity. In some studies, ESA administration to tumor-cell lines increased phosphorylation of ERK [56, 57]. However, ERK and AKT act downstream of multiple ligand/receptor complexes [58] and can be phosphorylated in response to changes in culture medium, pH, temperature [59], and bacterial lipopolysaccharide [60, 61], offering the possibility of false-positive results. Further, substances that can promote cell growth under certain conditions are found in Epo formulations, such as in serum or in bovine-serum albumin protein preparation used to stabilize rHuEpo. These substances can also induce signaling comparable to that observed in some ESA experiments with cell lines [59].

Demonstrating phosphorylation of the downstream signaling molecules JAK2 and STAT5 is more specific to EpoR activation than demonstrating ERK and AKT phosphorylation. However, investigators have not always evaluated phosphorylation of JAK2 and STAT5. For example, Gewirtz et al. [56] and Lester et al. [57] reported that rHuEpo stimulated ERK phosphorylation in MCF-7 breast-cancer cells, but did not evaluate effects on phosphorylation of STAT5 or JAK2. In addition, other investigators have been unable to duplicate those ERK results [18, 21], and control experiments suggest that the positive effects were likely due to contaminants or to medium changes, emphasizing the need for controls in such studies to rule out false-positive effects. Additionally, since EpoR requires accessory factors (e.g., JAK2) for transport to the cell surface and for signaling [38], examination of the phosphorylation status of JAK2 or STAT5 would be informative. However, such experiments are difficult to perform and negative results have been reported. Therefore, other indirect methods have been used. For example, inhibitors of JAK2 (e.g., AG490) were used to examine potential EpoR-mediated effects with positive results reported. An assumption of such experiments is that the inhibitors are specific to EpoR. Though AG490 inhibits multiple kinases, it has little/no inhibitory effect on JAK2 in vitro and, therefore, would not impact the EpoR signaling pathway [62]. Thus, data using such JAK2 inhibitors must be interpreted with caution.

2. Receptor signaling must occur in response to relevant ligand concentrations.

In several studies, there was increased proliferation of tumor-cell lines on Epo addition, but with supraphysiological Epo levels that were 10- to 1,000-fold greater than the maximum plasma level observed in patients receiving approved Epo doses. For example, one report described how culturing cells in a medium containing 250,000 mU/mL rHuEpo resulted in EpoR-mediated tyrosine phosphorylation [63]. With administration of such high Epo doses, false-positive results can occur due to non-specific vehicle effects or low-level growth-promoting contaminants.

3. Enough receptor must be present to provide a meaningful response.

Studies suggest that EpoR-protein levels are very low in tumors and tumor-cell lines. As one example, MCF-7 breast-cancer cells have a total of only 100 EpoR dimers/cell vs the ∼10,000 receptors (surface plus cytoplasmic EpoR) seen in erythroid progenitors [21] but have been reported to respond to Epo addition. Given the inefficient transport of EpoR to the cell surface, the number of EpoR molecules available for ligand binding on MCF-7 cells would be very low. Accordingly, there is conflicting data with MCF-7 cells as Laugsch et al. [18] and other groups [21] were unable to confirm reports that rHuEpo stimulated proliferation of MCF-7 and other cell lines where EpoR levels are very low. Another confounding factor is that non-hematopoietic cells and tumor cells may not have the same signaling networks found in erythroid-progenitor cells or may have constitutive activation of pathways. Either can render the cells nonresponsive to growth factor challenge. Thus, cells may not respond to Epo, even with high-level EpoR expression. Consistent with this, forced overexpression of EpoR in some factor-dependent myeloid cells or solid-tumor cell lines does not result in Epo responsiveness [64, 65].

Apart from cell culture work, another approach is to employ animal experiments. Studies using rodent-tumor models have indicated that ESA administration alone does not stimulate tumor proliferation [49]. In addition, ESAs could hypothetically inhibit the effects of concurrently administered anticancer therapy. One report indicated that rHuEpo alone had no effect on MCF-7 cell growth in animals, but that tumor size increased in trastuzumab-treated animals [66]. However, as noted above, MCF-7 cells express minimal levels of EpoR and in studies by other groups, treating MCF-7 cells with Epo failed to interfere with the antiproliferative and/or cytotoxic effects of either bevacizumab or paclitaxel [67, 68].

In summary, demonstrating functional EpoR requires proof that cell-surface EpoR molecules exist in adequate concentration and that downstream signaling occurs in response to relevant ligand concentrations. In addition, tests must be performed using positive and negative controls.

## *EpoR* mutations/amplification and *Epo* overexpression

Mutations in genomic-coding sequences of receptor genes can be tumorigenic or alter the potential for receptors to signal. These include mutations that constitutively activate a receptor, increase gene-copy number (and ultimately gene-expression level of a receptor), or alter factors that regulate protein processing, which can lead to receptor overproduction. These mutations are usually identified through specific DNA sequencing of the gene on genomic clones, through sequencing of mRNAs encoding the protein (using PCR/cloning), or through complete genome/transcriptome sequencing using next-generation sequencing methodologies.

### *EpoR* mutations

A mutation in murine *EpoR*, R129C, was identified that results in EpoR dimerization and constitutive activation. In mice, this mutation causes erythroid and granulocyte/monocyte colony-expansion but not expansion of other hematopoietic lineages, indicating that nonmyeloid-cell types have a limited response to activated EpoR [69–71].

To date, no *EpoR* mutations have been reported in any solid tumors. *EpoR*-activating mutations have not been observed in erythroleukemias [72, 73], and erythroleukemias are not associated with alterations to chromosome 19 where the *EpoR* gene is located [72]. *EpoR*-hyperactivity truncation mutations cause erythrocytosis in humans [74, 75], but not leukemias or tumors [72, 76]. When Gonda et al. [72] screened ten human cases of erythroleukemia, no *EpoR*-gene mutations were found.

In summary, though EpoR hyperactivity can cause erythrocytosis and erythroleukemia in mice, no evidence to date suggests that *EpoR* hyperactivating mutations in humans increase cancer risk or enhance tumor growth.

### *EpoR* gene amplification

Amplification of certain oncogenic receptors (e.g., *HER2* or *EGFR*) is characteristic of some aggressive tumors. Amplification of the *EpoR* gene has been detected in some cell lines derived from AML, CML, and erythroleukemia patients (e.g., UT-7 and TF-1) consistent with their myeloid origin [77, 78]. This amplification may provide these cells with a growth advantage when treated with ESAs. However, *EpoR*-gene amplification is not a characteristic of solid tumors. A gene-amplification analysis of 1,083 solid tumors showed that *EpoR*-gene amplification was rare; overall, the *EpoR*-gene frequency was similar to other non-oncogenes [47].

### Epo overexpression

Another suggested theory is that long–term exposure to elevated levels of Epo might induce tumors. However, this theory is not supported by patients with mutations resulting in lifetime elevated-Epo levels. Increased cancer incidence is not observed in patients with Chuvash polycythemia who have increased levels of endogenous Epo [79, 80]. Similarly, people living at high altitude (>3,000 ft) with chronically elevated endogenous Epo have no increase in tumor incidence, tumor mortality, or tumor recurrence compared with those living at sea level [81]. In summary, elevated levels of endogenous Epo do not appear to increase the incidence of cancer.

## Angiogenesis

As an alternative to direct stimulation of tumors via resident receptors, tumor growth may be increased if an activated receptor stimulates angiogenesis. During angiogenesis, new vessels arise from existing vessels through endothelial branching, sprouting, migration, and proliferation [82, 83]. One hypothesis is that Epo or exogenous ESAs could stimulate tumor growth by activating EpoR on endothelial cells to facilitate angiogenesis near tumors.

While several groups have reported detection of EpoR on endothelial cells, those results are controversial because of the same antibody non-specificity issues reported above for IHC and Western blotting. When a specific EpoR antibody was used to evaluate EpoR on endothelial cells [13], little EpoR protein was detected. Further, rHuEpo does not appear to bind to endothelial cells, and ESAs exerted no effect on endothelial cells in controlled experiments performed in vitro or in vivo [13, 84–86]. Studies in *EpoR*-null mutant mice expressing EpoR exclusively in the hematopoietic lineage have shown that the Epo–EpoR signaling pathway is not necessary for endothelial cell development [11]. In tumor-xenograft studies, no effect on angiogenesis was observed with ESAs [87–89]. In contrast to these data, there are positive results suggesting ESAs can stimulate angiogenesis. For example, Yamaji et al. [90] reported increased proliferation (as measured by thymidine incorporation) of brain-capillary endothelial cells following Epo addition but only when accompanied by a change in medium. Though one group noted that angiogenesis increases in chicken eggs treated with rHuEpo [91–93], there is no cross-species activity of human Epo in chickens [94]. These questionable studies highlight the importance of appropriate controls to aid in interpretation of the data.

## Conclusion

The presence of a specific cell-surface receptor can be detected using multiple methodologies with recommended standard procedures involving (1) receptor-specific antibodies, (2) binding of radiolabeled ligands (however, to be interpretable, the experiments must include both negative and positive controls and quantification of results to confirm that receptor levels correlate with known levels needed for function), and (3) confirmation of receptor size at the appropriate molecular mass (kilodaltons). Strategies and guidelines for these standard procedures have been published and should be followed [12, 15, 26].

Given the complexity of the issues involved, growth-factor receptor research in cancers should be evaluated in light of the techniques and controls used. Limitations in these techniques can lead to contradictory results as shown here regarding research on whether functional EpoR is present on non-erythroid cells.
